# Anterior-Posterior and Inter-Limb Lower Body Strength Asymmetry in Soccer, Basketball, Futsal, and Volleyball Players

**DOI:** 10.3390/medicina58081080

**Published:** 2022-08-10

**Authors:** Koulla Parpa, Marcos Michaelides

**Affiliations:** Sport and Exercise Science, UCLan University of Cyprus, Pyla 7080, Cyprus

**Keywords:** bilateral asymmetry, unilateral asymmetry, imbalance, sports

## Abstract

*Background and Objectives:* The purpose of this study was twofold: (1) to examine strength asymmetries in elite athletes of different sports and (2) to examine the magnitude of asymmetries between elite and u18 athletes. *Materials and Methods:* A total of 254 athletes participated in this study. For the first purpose of the study, the group consisted of adult professional male basketball players (*n* = 30), elite male (*n* = 30) and female soccer players (*n* = 20), male futsal players (*n* = 30), elite male goalkeepers (*n* = 22), and professional female volleyball players (*n* = 20). For the second purpose, male youth goalkeepers (*n* = 22), youth female (*n* = 20) and male soccer players (*n* = 30), and youth male basketball players (*n* = 30) were compared to the adult athletes of the same sport. Asymmetries were measured utilizing a Humac Norm and Rehabilitation device. Testing included three maximal concentric flexion and extension repetitions at an angular speed of 60°/s. The differences in asymmetries were assessed using ANOVA followed by an LS means post-hoc analysis. An independent samples *t*-test was used to identify the differences between adult and youth players. *Results:* It was indicated that none of the groups demonstrated asymmetries greater than 10%, other than the elite female soccer players and female volleyball players. Significant differences were demonstrated between youth and adult soccer players (males and females), with the adult groups demonstrating increased asymmetries. *Conclusions:* Special consideration should be given to female soccer players and volleyball players, as soccer and volleyball practice and competition at the professional level may induce greater lower -limb asymmetries. The isokinetic parameters can be useful for planning strength and conditioning interventions in order to reduce or prevent those imbalances. Additional unilateral and bilateral jumping testing is encouraged for the verification of imbalances.

## 1. Introduction

Many sports are characterized by asymmetric kinetic patterns and unilateral actions such as jumps, sprints, and changes in direction [1]. Research indicates that participating in team sports that mainly involve a particular side of the body entails asymmetrical changes in certain tissues [2]. Even though a certain degree of asymmetry in the human body and the predominance of one limb over the other is normal, unilateral loading over a long period may lead to the development of various degrees and modes of functional asymmetries [3]. Therefore, asymmetries are an adaptive consequence of a long-term, one-sided strain due to the technical elements performed during sports practice and games [3]. 

Lower limb asymmetry with regard to strength or power has been investigated in different sports [2] such as soccer [4], basketball [4,5,6], tennis [4], handball [5], and volleyball [6]. In addition, unilateral and bilateral lower body strength or power asymmetries have been investigated in young elite athletes [2,7], female soccer players [8,9], and soccer goalkeepers [10]. Concurrently, lower limb asymmetries have been investigated in athletes of different levels or divisions [11]. Unilateral and bilateral asymmetries have been commonly quantified with jumping-based tests [5,6,10] and isometric/isokinetic strength tests [2,7]. Lower limb asymmetry has been the subject of numerous investigations, as it has been identified as a potential factor contributing to impaired sports performance [12] and a higher risk of incurring injuries [13,14]. More specifically, research has indicated that as the strength asymmetry between the agonist and antagonist (knee extensors and flexors) increases, kicking inaccuracy also increases, as has been detected in soccer players [12]. Furthermore, with regard to injury risk, research has indicated that greater asymmetry during the pre-season period might reveal a potential weakness predisposing an athlete to injury [13]. Optimal concentric hamstring-to-quadricep isokinetic torque ratios have been reported to range between 0.5 and 0.75, while bilateral differences in muscular strength (torque) greater than 15% are considered abnormal and can be used as predictors of injury or indicators of incomplete rehabilitation programs [15]. Additionally, a 10–15% threshold of interlimb vertical jump height asymmetry has been considered the physiological norm in basketball and volleyball players [6]. 

Despite the aforementioned studies, evidence to the contrary does exist. Research is not unanimously clear that limb asymmetry is detrimental to performance, as some investigators suggest that sports asymmetries do not appear to carry an evident influence on athletic performance measures [16]. Research is also contradictory as to whether long-term participation in team sports such as soccer may increase the level of asymmetry [17]. In this regard, it has been indicated that players with a longer professional training age demonstrate a more balanced use of their lower extremities and cope with previously developed musculoskeletal asymmetries, which possibly reduces the injury risk [17]. Furthermore, research has demonstrated inconsistent asymmetries in volleyball players based on the speed of isokinetic testing [18] that was used to identify the asymmetries, as well as differences in testing modalities [19]. Additional studies have indicated significant differences between testing approaches in view of their sensitivity to detect inter-limb asymmetries, as well as the magnitude of inter-limb asymmetries in particular sports disciplines [4,8]. Regardless of the test selected, another consideration for asymmetry is how the data are reported. Some testing protocols report an average of three trials [20] while other studies determine the asymmetry from the best trial [11,21]. 

Consequently, due to the high inconsistency of findings and testing modalities in the literature [11,18,19,20,21], it is difficult to conclude the relationship between inter-limb asymmetries and participation in various sports disciplines. In addition, it is debatable whether the magnitude of these asymmetries is different in elite adult athletes compared to younger athletes with less exposure to the specific sport. Given the scarcity of data in this regard, the current investigation intended to use the same isokinetic test to identify asymmetries in athletes of different sports. 

Therefore, the purpose of this study was twofold: (1) to examine anterior-posterior and inter-limb strength asymmetries in elite male and female soccer players, professional male goalkeepers, professional male basketball players, male professional futsal players, and professional female volleyball players utilizing the same isokinetic test; and (2) to examine the magnitude of asymmetries between elite and u18 male soccer players, elite and u18 female soccer players, male professional and u18 basketball players, and male professional and u18 goalkeepers. It is hypothesized that long-term professional training would lead to greater limb asymmetries and imbalances. 

## 2. Materials and Methods

### 2.1. Participants 

A total of 254 professional and youth athletes participated in this study. More specifically, for the first purpose of the study, the analyzed group consisted of adult professional male basketball players (MBP) (*n* = 30, age = 27.23 ± 4.19 years), elite male (EMSP) (*n* = 30, age = 28.23 ± 4.95 years) and elite female soccer players (EFSP) (*n* = 20, age = 23.90 ± 3.78 years), male futsal players (MFP) (*n* = 30, age = 27.97 ± 4.34 years), elite male goalkeepers (EMGL) (*n* = 22, age = 25.77 ± 3.79 years), and professional female volleyball players (FVP) (*n* = 20, age = 25.35 ± 4.44 years). For the second purpose of the study, male youth goalkeepers (YMGL) (*n* = 22, age = 16.86 ± 1.08 years), youth female (YFSP) (*n* = 20, age = 15.15 ± 0.99 years) and male soccer players (YMSP) (*n* = 30, age = 17.20 ± 0.55 years), and youth male basketball players (YMBP) (*n* = 30, age = 15.20 ± 1.38 years) were compared to the adult athletes of the same sport. Athletes with a previous lower-body injury within the last six months were excluded from the study. Furthermore, youth players should have engaged in formal training sessions (3–4 sessions/week, ~90 min/session) and participated in nine-month competitive seasons over the entire training period of four years to be included in the study. It should be noted that all participants had prior experience with isokinetic testing. The testing was conducted during the in-season period and lasted approximately two weeks. All athletes were advised to abstain from heavy physical activity the day prior to testing, and the measurements were obtained between 9:00 and 15:00. Parents or legal guardians and the adult athletes provided written informed consent after receiving verbal and written information about the study’s procedures, associated risks, and benefits. The study was performed in accordance with the Declaration of Helsinki and was approved by the Ethics Committee of the University (reference number STEMH 541). 

### 2.2. Procedures

Anthropometric data were recorded before testing muscular strength and asymmetries. Body stature was measured using a wall stadiometer (Leicester; Tanita, Japan), while a leg-to-leg bioelectrical impedance analyzer (BC418MA; Tanita) was utilized to measure body composition. The lower limb asymmetries were measured utilizing a Humac Norm and Rehabilitation device (CSMI, Stoughton, MA, USA) according to the methods described by previous investigators [22]. Prior to the isokinetic testing, athletes performed a 5 min self-paced warm-up on a mechanically braked cycle ergometer (Monark 894 E Peak Bike, Weight Ergometer, Vansbro, Sweden). The testing began with the athletes sitting with their thighs at an angle of 85° to the trunk, while the axis of rotation of the dynamometer was aligned with the lateral epicondyle of the knee joint. The range of motion at the knee joint was 100°. The upper body, thigh, and ankle were fixed using the machine’s straps. Once the players were appropriately positioned on the isokinetic device, they performed five sub-maximal repetitions of concentric knee flexion and extension for further familiarization. Testing included three maximal concentric flexion and extension repetitions at an angular speed of 60°/s. A standard rest interval of 1 min was included between the individual sets and verbal feedback was provided throughout the tests. Appropriate calibration and gravity correction were performed before the testing by the same experienced tester.

### 2.3. Statistical Analysis

IBM^®^ SPSS^®^ Statistics, version 26.0, for Windows (SPSS Inc., Chicago, IL, USA ) was utilized to analyze the results. Means and standard deviations were calculated for all the parameters. The homogeneity of variance and normality assumptions were verified using Brown and Forsythe’s and Shapiro–Wilk tests, respectively. The differences in asymmetries between the athletes of different sports were assessed using a one-way analysis of variance (ANOVA) followed by an LS means post-hoc analysis to identify which sports differed. The effect size was estimated with partial eta-squared (η^2^). The effect sizes were interpreted as follows: large (partial η^2^ ≥ 0.14), medium (partial η^2^ ≥ 0.06), and small (partial η^2^ ≥ 0.01) [23]. Furthermore, an independent samples t-test was used to identify the differences in asymmetries between elite and youth players of the same sport. Cohen’s d was calculated to determine the effect size. Effect sizes were interpreted as small (0.2–0.4), medium (0.5–0.7), and large (0.8–1.4) [24]. For all statistical analyses, significance was accepted at *p* < 0.05.

## 3. Results 

The anthropometric and body composition parameters are presented in Table 1, and the isokinetic torques are presented in Table 2. One-way analysis of variance (ANOVA) indicated statistically significant differences in the right leg (*F*(5,146) = 2.95, *p* < 0.05, partial η^2^ = 0.09) and left leg (*F*(5,146) = 2.64, *p* < 0.05, partial η^2^ = 0.08) ratios based on the sport. Furthermore, one-way ANOVA demonstrated statistically significant differences between the right and left extensors (quadriceps) (*F*(5,146) = 4.26, *p* < 0.05, partial η^2^ = 0.13), as well as right and left flexors (hamstring) (*F*(5,146) = 5.00, *p* < 0.05, partial η^2^ = 0.15) based on the sport. Further analysis indicated that the right leg ratio was significantly different between EFSP and FVP (*p* < 0.05), as well as between MFP and FVP (*p* < 0.05) (Table 3). Regarding the left leg ratio, the only significant difference was indicated between the EMSP and FVP (*p* < 0.05). Considering the asymmetries between the right and left extensors (quadriceps), differences were demonstrated between MBP and EFSP (*p* < 0.05), as well as between EFSP and EMGL (*p* < 0.05) (Table 3). In addition, considering the asymmetries between the right and left flexors (hamstring), differences were indicated between MBP and FVP (*p* < 0.05), between MFP and EMGL (*p* < 0.05), and between EMSP and EMGL (*p* < 0.05) (Table 3). Concurrently, asymmetries between the right and left flexors were indicated between EMGL and FVP (*p* < 0.05; Table 3). 

Regarding asymmetries between youth and adult professional players of different sports, the results indicated no significant differences in the right and left leg ratios between YMSP and YMGL. On the contrary, YMGL had significantly greater imbalances for the hamstring muscle group (*t*(42) = –2.73, *d* = 0.86, *p* < 0.05). Even though the bilateral asymmetries were greater for YMGL, the results were within normal ranges (below 10%). When comparing YFSP and adult EFSP, significant differences were evident in the right leg ratio (*t*(38) = 3.026, *d* = 0.98, *p* < 0.05), as well as the quadricep asymmetries (*t*(38) = 3.54, *d* = 1.12, *p* < 0.05), with the adult EFSP demonstrating significantly greater bilateral (quadriceps) asymmetries (12%). Regarding the EMSP and YMSP, significant differences were indicated in both the right (*t*(58) = 2.64, *d* = 0.71, *p* < 0.05) and left (*t*(58) = 3.40, *d* = 0.89, *p* < 0.05) leg ratios. In addition, significant differences between EMSP and YMSP were identified between the right and left quadriceps (*t*(58) = 2.38, *d* = 0.62, *p* < 0.05) and right and left hamstring muscles (*t*(58) = 2.43, *d* = 0.63, *p* < 0.05). Regarding YMBP and adult MBP, the only significant difference was identified between the right and left quadriceps, with the youth players demonstrating significantly greater bilateral asymmetries for the quadricep muscle group (*t*(58) = –2.60, *d* = 0.68, *p* < 0.05). It should be noted that even though the YMBP demonstrated significantly greater bilateral asymmetry for the quadriceps, the asymmetry was below 10%. Youth players’ asymmetries and imbalances are presented in Table 4. Differences between the youth and adult groups are presented in Table 5. 

## 4. Discussion 

The present study was the first to compare lower limb anterior-posterior and inter-limb asymmetry in professional soccer players, goalkeepers, basketball players, futsal players, and volleyball players, utilizing the same isokinetic test. Furthermore, those asymmetries were compared between elite and u18 male soccer players, elite and u18 female soccer players, professional and u18 basketball players, and professional and u18 goalkeepers. Undoubtedly, performing isokinetic testing does not replicate angular velocities of many functional activities [25]. However, the isokinetic testing in this study was performed at slower speeds (60°/s) in order to determine the maximum isokinetic strength (torque) and deficits as recommended by previous investigators [26]. Our results indicated that FVP had a significantly lower right ratio (H/Q) than EFSP and a significantly lower left leg ratio (H/Q) than EMSP. Considering the right and left leg ratios, the athletes of different sports demonstrated ratios between 70% and 80%. Concurrently, regarding inter-limb asymmetries, even though some significant differences were demonstrated between the athletes of different sports, none of the groups demonstrated values greater than 10%, other than the EFSP and FVP (Table 3). Furthermore, the youth athletes’ right and left leg ratios were indicated to be between 65% and 74%, with the YFSP demonstrating the lowest ratios. The ratios of youth and adult professional athletes were similar to those reported in the active young population [12]. Additionally, none of the youth groups demonstrated inter-limb asymmetries, even though the YMGL and YMBP demonstrated significantly greater imbalances than the adult professional players. Significant differences were demonstrated between youth and adult soccer players (female and male athletes), with the adult groups demonstrating increased asymmetries. Based on our results, it may be suggested that long-term and intensive activities in sports such as soccer and volleyball, which are characterized by intermittent and irregularly alternating cyclic and acyclic movements, may lead to asymmetry and dominance of one leg. On the other hand, the specific volume and intensity of the physical load for goalkeepers and basketball players may lead to reductions in asymmetries that may be related to pre-existing limb preference. 

Specifically, our results demonstrated that sport-specific demands could lead to the development of some degree of asymmetric adaptations for specific sports, such as female soccer, where athletes tend to use one extremity more than the other. These asymmetries in female soccer players may have arisen due to differences in training experience [17], level of sports practice, repetitive asymmetrical sport-specific demands, or injury history with incomplete recovery [27]. It should be noted that the asymmetry was below 15%, which is not considered problematic [15]. However, it was over 10%, which has been proposed as a threshold for athletes [28]. Additionally, given the important variability (mean = 12.80 ± 12.04%) shown between the right and left quadriceps of the EFSP, it is essential to evaluate these players individually. The quadricep asymmetry in female soccer players should not be ignored, considering the higher rate of specific injuries such as anterior cruciate ligament ruptures in female compared to male athletes [29]. Furthermore, an inter-limb imbalance is frequently greater in female compared to male athletes in relation to strength, coordination, and postural control [30].

Regarding the male soccer players, YMSP and adult EMSP did not demonstrate inter-limb asymmetries. While some studies have demonstrated that male soccer players possess various muscle strength asymmetries [31], mainly attributed to preferred sidedness in executing most unilateral soccer skills, our results are parallel to those that failed to confirm significant bilateral leg strength asymmetries in male soccer players [32]. Of note, even though no significant bilateral strength asymmetries were demonstrated in male soccer players, the overall magnitude of asymmetry was greater in male soccer and futsal players compared to male basketball players and goalkeepers. Similarly, Šarabon and colleagues (2020) [4] demonstrated that inter-limb strength asymmetries were larger in soccer players than in basketball and tennis players. 

The FVP in this study also demonstrated a higher degree of asymmetry (Q:Q = 9.65 ± 6.20% and H:H = 11.05 ± 5.71%) than the rest of the groups. These asymmetries may be partially explained by the high volume of repetitive jumping and landing with a single or double leg [33]. Our results are in agreement with the results of Kalata and colleagues (2020) [2] who also demonstrated similar values of bilateral asymmetry (11.74 ± 7.41%) in volleyball players. Furthermore, similar to our findings, Hadzic and colleagues (2010) [34] indicated that the H:Q observed ratios are lower in male volleyball players than in soccer players, which may be attributed to the lower sprinting demands of volleyball compared to soccer. 

Regarding youth athletes, the YMGL and YMBP demonstrated greater bilateral asymmetries than adults of the same sport, but the values were within the normal range. This finding suggests that longer professional training may result in the more balanced use of lower extremities and a reduction in previous musculoskeletal asymmetries. On the contrary, a longer professional training age in sports such as soccer may result in increased asymmetries based on the results of our study. When comparing YFSP and adult EFSP, significant differences were evident in the right leg ratio (H:Q), as well as the quadricep asymmetries (Q:Q), with elite adult female soccer players demonstrating significantly greater bilateral (quadriceps) asymmetries (12%). Regarding the EMSP and YMSP, significant differences were indicated in both the right and left leg ratios. In addition, significant differences between the EMSP and YMSP were identified between the right and left quadriceps and right and left hamstring muscles. The aforementioned findings do not agree with the findings of Fusekis and colleagues (2010) [17] who suggested that long-term participation in team sports such as soccer may decrease the level of asymmetry. 

## 5. Conclusions

This study incorporated essential information including team sports, such as volleyball, which have been little studied and compared with other sports. Furthermore, this study indicated that even though asymmetric kinetic patterns and unilateral actions characterize some sports, it does not necessarily mean that they will result in anterior-posterior or inter-limb asymmetries. Special consideration should be given to soccer players, predominantly female soccer players. Furthermore, attention should be placed on female volleyball players, as they also demonstrated some asymmetry. It is suggested that more frequent monitoring of the asymmetries should be conducted utilizing additional unilateral and bilateral testing such as countermovement jump, squat, and drop jumps for the verification of those imbalances as well as the association of those imbalances with physical performance and increased injury risk. 

## 6. Limitations

Despite the presented results, this study comes with limitations. There were no differences in playing positions due to the limited number of athletes presented in each sport. Consequently, we strongly recommend that future studies collect information based on playing positions by incorporating a much larger sample size. Furthermore, the unilateral load presented in sports such as volleyball and basketball could negatively affect not only the lower limbs but also the upper limbs and trunk muscles in terms of symmetries. Therefore, future research is encouraged to focus on assessing asymmetries and imbalances of the lower body as well as the upper limbs and trunk muscles. Lastly, future research should be conducted by defining limbs as “dominant” and “non-dominant, not as right and left, as in our study. That said, it does not always mean that the dominant limb will be the superior limb regarding performance [35,36].

## Figures and Tables

**Table 1:** Anthropometric and body composition parameters (mean ± SD) by sport.

Sport	*n*	Age (Years)	Height (cm)	Weight (kg)	Body Fat (%)
MBP	30	27.23 ± 4.19	192.80 ± 7.71	92.58 ± 9.79	12.84 ± 3.14
EMSP	30	28.23 ± 4.95	177.53 ± 4.69	74.64 ± 5.29	11.19 ± 3.11
EFSP	20	23.90 ± 3.78	165.00 ± 4.26	59.23 ± 6.57	20.06 ± 3.96
MFP	30	27.97 ± 4.34	174.33 ± 5.45	78.19 ± 12.58	16.61 ± 5.17
EMGL	22	25.77 ± 3.79	189.27 ± 3.70	86.77 ± 5.59	12.16 ± 2.69
FVP	20	25.35 ± 4.44	176.04 ± 7.41	71.46 ± 8.35	23.49 ± 4.49
YMGL	22	16.86 ± 1.08	182.41 ± 5.15	75.34 ± 8.63	13.61 ± 3.21
YFSP	20	15.15 ± 0.99	160.75 ± 3.83	52.36 ± 4.70	23.07 ± 2.10
YMSP	30	17.20 ± 0.55	176.07 ± 6.27	66.30 ± 6.54	8.42 ± 4.08
YMBP	30	15.20 ± 1.38	179.92 ± 7.54	73.01 ± 8.46	17.99 ± 3.76

Note: MBP: Male Basketball Players; EMSP: Elite Male Soccer Players, EFSP: Elite Female Soccer Players; MFP: Male Futsal Players; EMGL: Elite Male Goalkeepers; FVP: Female Volleyball Players; YMGL: Youth Male Goalkeepers; YFSP: Youth Female Soccer Players; YMSP: Youth Male Soccer Players; and YMBP: Youth Male Basketball Players.

**Table 2:** Peak isokinetic torque (Nm) (mean ± SD) by sport.

Sport	*n*	Right Quadriceps (Nm)	Left Quadriceps (Nm)	Right Hamstring (Nm)	Left Hamstring (Nm)
MBP	30	267.77 ± 35.17	263.03 ± 36.64	195.73 ± 29.70	195.20 ± 26.44
EMSP	30	216.10 ± 28.33	210.13 ± 33.26	164.73 ± 22.87	167.10 ± 27.60
EFSP	20	138.75 ± 24.95	145.00 ± 28.46	106.75 ± 18.74	102.40 ± 19.07
MFP	30	211.47 ± 38.46	213.07 ± 31.19	161.60 ± 24.73	157.97 ± 17.83
EMGL	22	264.41 ± 28.60	261.45 ± 29.69	192.95 ± 23.10	193.18 ± 22.85
FVP	20	168.45 ± 30.21	161.85 ± 28.58	109.00 ± 19.83	110.50 ± 18.75
YMGL	22	228.59 ± 26.13	228.36 ± 34.59	162.82 ± 21.49	163.14 ± 17.24
YFSP	20	127.95 ± 12.94	125.05 ± 15.17	84.35 ± 14.29	86.55 ± 12.19
FMSP	30	210.77 ± 30.60	210.13 ± 30.54	146.67 ± 19.23	147.90 ± 20.62
YMBP	30	199.80 ± 35.70	198.73 ± 32.54	143.87 ± 23.62	145.47 ± 26.67

Note: MBP: Male Basketball Players; EMSP: Elite Male Soccer Players, EFSP: Elite Female Soccer Players; MFP: Male Futsal Players; EMGL: Elite Male Goalkeepers; FVP: Female Volleyball Players; YMGL: Youth Male Goalkeepers; YFSP: Youth Female Soccer Players; YMSP: Youth Male Soccer Players; and YMBP: Youth Male Basketball Players.

**Table 3:** Intra- and inter-limb strength asymmetries (mean ± SD) in different sports.

Sport	Right Leg Ratio (H/Q)	95% CI for Mean	Left Leg Ratio (H/Q)	95% CI for Mean	Bilateral Asymmetries % (RQ:LQ)	95% CI for Mean	Bilateral Asymmetries % (RH:LH)	95% CI for Mean
MBP (*n* = 30)	74.07 ± 13.41	69.06–79.07	75.53 ± 14.54	70.10–80.96	5.93 ± 3.78 #	4.52–7.34	5.33 ± 3.67 **	3.97–6.70
EMSP (*n* = 30)	76.80 ± 10.61	72.84–80.76	80.23 ± 11.34 ^	76.00–84.47	8.37 ± 5.86	6.18–10.55	9.33 ± 8.27 ^^^	6.24–12.42
EFSP (*n* = 20)	79.05 ± 18.65 *	70.32–87.78	73.20 ± 13.32	66.97–79.43	12.80 ± 12.04 #^^	7.17–18.43	8.65 ± 7.19	5.28–12.02
MFP (*n* = 30)	78.27 ± 13.97 +	73.05–83.48	75.07 ± 10.23	71.25–78.88	7.93 ± 6.84	5.38–10.49	9.53 ± 7.78 ++	6.63–12.44
EMGL (*n* = 22)	73.45 ± 9.34	69.31–77.60	74.18 ± 8.19	70.55–77.81	4.36 ± 3.02 ^^	3.03–5.70	3.32 ± 2.84 ++^^^ ^X^	2.06–4.58
FVP (*n* = 20)	65.75 ± 11.53 *+	60.35–71.15	68.70 ± 7.97 ^	64.97–72.43	9.65 ± 6.20	6.75–12.55	11.05 ± 5.71 ** ^X^	8.38–13.72

Note: MBP: Male Basketball Players; EMSP: Elite Male Soccer Players; EFSP: Elite Female Soccer Players; MFP: Male Futsal Players; EMGL: Elite Male Goalkeepers; FVP: Female Volleyball Players; RQ:LQ: Right Quadriceps:Left Quadriceps difference in %; RH:LH: Right Hamstring:Left Hamstring difference in % H/Q: hamstring to quadriceps ratio; * *p* < 0.05 denotes significant difference between EFSP and FVP, + *p* < 0.05 denotes significant difference between MFP and FVP. ^ *p* < 0.05 denotes significant difference between EMSP and FVP; # *p* < 0.05 denotes significant difference between MBP and EFSP; ^^ *p* < 0.05 denotes significant difference between EFSP and EMGL; ** *p* < 0.05 denotes significant difference between MBP and FVP; ++ *p* < 0.05 denotes significant difference between MFP and EMGL; ^^^ *p* < 0.05 denotes significant difference between EMSP and EMGL; and ^X^ *p* < 0.05 denotes significant difference between EMGL and FVP.

**Table 4:** Intra- and inter-limb strength asymmetries (mean ± SD) of youth players of different sports.

Sport	Right Leg Ratio (H/Q)	Left Leg Ratio (H/Q)	Bilateral Asymmetries % (RQ:LQ)	Bilateral Asymmetries % (RH:LH)
YMGL (*n* = 22)	71.68 ± 9.11	72.59 ± 9.97	6.41 ± 4.91	6.18 ± 3.81
YFSP (*n* = 20)	65.75 ± 6.21	69.40 ± 5.93	2.90 ± 3.46	5.55 ± 4.25
YMSP (*n* = 30)	70.23 ± 8.56	71.07 ± 9.47	5.50 ± 3.01	5.37 ± 3.40
YMBP (*n* = 30)	71.67 ± 11.17	73.77 ± 11.22	9.67 ± 6.90	7.60 ± 5.42

Note: YMGL: Youth Male Goalkeepers; YFSP: Youth Female Soccer Players; YMSP: Youth Male Soccer Players; YMBP: Youth Male Basketball Players; H/Q: hamstring to quadriceps ratio; RQ:LQ: Right. Quadriceps:Left Quadriceps difference in %; and RH:LH: Right Hamstring:Left Hamstring difference in %.

**Table 5:** Differences between the youth and adult groups.

Sport	Variables	Mean Difference	*p*	Cohen’s d	95% CI Lower -Upper	
EMGL-YMGL	Right leg ratio (H/Q)	1.77	0.53		−3.84	7.39
	Left leg ratio (H/Q)	1.59	0.57		−3.96	7.14
	Bilateral asymmetries % (RQ:LQ)	−2.045	0.11		−4.54	0.45
	Bilateral asymmetries % (RH:LH)	−2.86	0.007	d = 0.86	−4.91	−0.82
EFSP-YFSP	Right leg ratio (H/Q)	13.30	0.006	d = 0.98	4.21	22.39
	Left leg ratio (H/Q)	3.80	0.25		−2.90	10.50
	Bilateral asymmetries % (RQ:LQ)	9.90	0.002	d = 1.12	4.09	15.71
	Bilateral asymmetries % (RH:LH)	3.10	0.11		−0.71	6.91
EMSP-YMSP	Right leg ratio (H/Q)	6.57	0.01	d = 0.71	1.58	11.55
	Left leg ratio (H/Q)	9.17	0.001	d = 0.89	3.77	14.57
	Bilateral asymmetries % (RQ:LQ)	2.87	0.02	d = 0.62	0.44	5.29
	Bilateral asymmetries % (RH:LH)	3.97	0.02	d = 0.63	0.66	7.27
MBP-YMBP	Right leg ratio (H/Q)	2.40	0.45		−3.98	8.78
	Left leg ratio (H/Q)	1.77	0.60		−4.94	8.48
	Bilateral asymmetries % (RQ:LQ)	−3.73	0.013	d = 0.68	−6.62	−0.84
	Bilateral asymmetries % (RH:LH)	−2.27	0.063		−4.66	0.13

Note: EMGL: Elite Male Goalkeepers; YMGL: Youth Male Goalkeepers; EFSP: Elite Female Soccer Players; YFSP: Youth Female Soccer Players; EMSP: Elite Male Soccer Players; YMSP: Youth Male Soccer Players; MBP: Male Basketball Players; YMBP: Youth Male Basketball Players; H/Q: hamstring to quadriceps ratio; RQ:LQ: Right Quadriceps:Left Quadriceps difference in %; and RH:LH: Right Hamstring: Left Hamstring difference in %.

## Data Availability

Data can be obtained by contacting the lead author K.P.
